# Performance of calf circumference in identifying sarcopenia in older patients with chronic low back pain: a retrospective cross-sectional study

**DOI:** 10.1186/s12877-024-05263-z

**Published:** 2024-08-10

**Authors:** Hee Jung Kim, Ji Young Kim, Shin Hyung Kim

**Affiliations:** Department of Anesthesiology and Pain Medicine, Anesthesia and Pain Research Institute, Yonsei University College of Medicine, 50 Yonsei-ro, Seodaemun-gu, Seoul, 03722 Republic of Korea

**Keywords:** Calf circumference, Sarcopenia, Older patients, Chronic low back pain

## Abstract

**Background:**

Calf circumference is currently recommended as a case-finding marker for sarcopenia, but its usefulness has not been determined in chronic pain conditions. Therefore, the present study aimed to evaluate the predictive performance of calf circumference in diagnosing sarcopenia in older patients with chronic low back pain.

**Methods:**

Ambulatory adult patients aged ≥ 65 years with chronic low back pain were enrolled. A diagnosis of sarcopenia was established based on the criteria outlined by the Asian Working Group for Sarcopenia in 2019. Patient demographics, pain-related factors, clinical factors, and sarcopenia-related measurements were compared between non-sarcopenic and sarcopenic patients. Linear regression analysis was used to evaluate the correlation of calf circumference with muscle mass, strength, and physical performance. Also, a receiver operating characteristic curve analysis for calf circumference in predicting sarcopenia was conducted; and area under the curve (AUC) values, along with their corresponding 95% confidence intervals (CI), were calculated.

**Results:**

Data from 592 patients were included in the analysis. Eighty-five patients were diagnosed with sarcopenia (14.3%), 71 of whom had severe sarcopenia (11.9%). A higher prevalence of sarcopenia was observed in female patients (9.0% vs. 16.7%, *p* = 0.016). After adjusting for age, BMI, and comorbidities, calf circumference correlated positively with muscle mass but not with muscle strength and physical performance. The AUC values for sarcopenia were 0.754 (95% CI = 0.636–0.871, *p* = 0.001) in males and 0.721 (95% CI = 0.657–0.786, *p* < 0.001) in females. The cut-offs for calf circumference in predicting sarcopenia were 34 cm (sensitivity 67.1%, specificity 70.6%) in males, and 31 cm (sensitivity 82.5%, specificity 51.5%) in females.

**Conclusions:**

Even though sex differences in its predictive value for sarcopenia should be considered, our findings suggest that calf circumference can be used as an indicator for predicting muscle mass and may serve as a potential marker for identifying sarcopenia in older patients with chronic low back pain.

## Background

Sarcopenia is currently defined as the decline in skeletal muscle mass and strength that occurs with advancing age and is often accompanied by diminished physical performance in its severe form [1, 2]. Sarcopenia is associated with adverse health outcomes, including increased risk of falls and fractures, higher rates of hospitalization, and elevated mortality risk [1, 3]. This condition is an increasing problem in our aging society; thus, sarcopenia prevention, treatment, and rehabilitation have become significant public health concerns when considering the economic and societal burden of sarcopenia. [3, 4].

Chronic low back pain (CLBP) is one of the most common and major disabling health conditions among older adult populations [5]. The prevalence of sarcopenia among older patients with CLBP seems to be somewhat higher than in patients without pain [6]. Also, sarcopenia is associated with poor CLBP treatment outcomes [6, 7]. Therefore, early identification of older patients at risk of sarcopenia is important for those with CLBP.

In the Asian Working Group for Sarcopenia 2019 (AWGS 2019) guidelines, calf circumference is recommended as an anthropometric measurement for identifying sarcopenia, facilitating early detection in older adults [1]. The role of calf circumference in the diagnosis algorithm for sarcopenia is supported by validation reports [8–13]. Calf circumference demonstrated a positive correlation with skeletal muscle mass assessed through dual-energy X-ray absorptiometry [8–12] and was also significantly associated with both muscle strength and physical performance [12]. However, these results were obtained from samples of the community-dwelling older population [12]. In recent reports, calf circumference showed promise for the screening for sarcopenia in subgroups with several comorbidities such as stroke, Parkinson’s disease, and diabetes [14–16]. However, the usefulness of calf circumference as a screening marker for sarcopenia has not been investigated in older patients with symptomatic degenerative lumbar spinal disease.

Accordingly, the aims of this study were to determine calf circumference cut-off values for sarcopenia prediction in older patients with CLBP and to evaluate its diagnostic performance using AWGS 2019 criteria. Also, the relationship between calf circumference and skeletal muscle mass, muscle strength, and physical performance was investigated in this population.

## Methods

### Study population

This study received approval from the Institutional Review Board of Yonsei University Health System, Seoul, Republic of Korea (IRB No. 4-2024-0094). In our previous studies, we have observed that low handgrip strength and high fat infiltration of paraspinal muscles resulted in poor treatment outcomes in older patients with CLBP [17, 18]. Therefore, in 2022, we began sarcopenia screening and diagnosis for older patients with chronic pain at their initial visit to our pain clinic. The present study employs a retrospective cross-sectional observational design. Specifically, it is a retrospective audit of CLBP patients who completed sarcopenia assessment based on the AWGS 2019 diagnostic protocol. Patients who visited our clinic seeking treatment for low back pain from January to December 2022 were enrolled in the study. Based on the patho-anatomical approach of CLBP used for confirmation [19], adult patients aged 65 years and above diagnosed with degenerative lumbar spinal disease, such as spondylolisthesis, herniated disc, spinal/foraminal stenosis, and facet joint arthropathy, confirmed by radiological evaluation within one year from the date of initial visit were included. Pain duration of three months or longer was used to define chronicity. Non-ambulatory patients or patients with severe cognitive impairment that precluded completion of the sarcopenia assessment protocol were excluded. Patients with abnormal calf asymmetry with a difference in circumference greater than 2.0 cm between calves [20] or pitting edema of the lower limbs were excluded. To assess lower limb pitting edema, visual inspection for swelling or skin changes, gentle palpation to assess skin indentation, and observation for persistence of indentation after pressure release were conducted. In addition, patients with incomplete medical records for this study were excluded.

#### Sarcopenia assessment

All measurements followed standard protocols for each measurement based on AWGS 2019 recommendations [1]. An independent nurse practitioner experienced in comprehensive geriatric assessment conducted all measurements throughout the study period. Calf circumference was measured at the widest part of both calves using a non-elastic tape to capture the maximum value. Patients were instructed to stand with their feet shoulder-width apart to ensure equal distribution of body weight. The tape was applied snugly but without compressing the calf and was positioned flat on the skin and parallel to the floor. After measuring each calf twice, an average circumference was recorded. Handgrip strength (HGS) was assessed by conducting three measurements on each hand using a Smedley-type dynamometer (EH101; CAMRY, Guangdong, China). Patients were instructed to stand with their elbows fully extended and to exert a maximum-effort isometric contraction while squeezing the dynamometer. The highest reading obtained from three measurements on each hand was recorded, and the maximum value from either hand was utilized for analysis. Appendicular skeletal muscle mass (ASM) was measured using a bioelectrical impedance analysis (BIA) device (Inbody H20N, InBody Co., Ltd., Seoul, Korea). Participants were instructed to undergo BIA measurements in the morning on an empty stomach to standardize body water distribution, ensuring they emptied their bladder and bowels and refrained from physical activities, showering, sauna use, or any activities affecting body moisture beforehand. Skeletal muscle mass index (SMI) was calculated by dividing ASM by the square of the patient’s height. A short physical performance battery (SPPB) was conducted, and its subtest scores and timings were determined. The SPPB consists of three subsets including static balance, gait speed, and chair sit-to-stand test [21]. To evaluate static balance, patients were instructed to maintain three standing postures of increasing difficulty, feet-together, semi-tandem, and full-tandem stance, for up to 10 s each. Patients were timed until movement or until 10 s had elapsed. For the gait speed test, patients walked at their comfortable pace across a 4-meter distance, and the average time for two trials was recorded. To assess chair sit-to-stand time, patients crossed their arms over their chests and, as quickly as possible, performed five stands from a standard chair. The time taken to complete the five sit-to-stand tasks was recorded. Each of the three subtests was scored on a scale from 0 to 4; the total score, ranging from 0 to 12, was the sum of these subtest scores.

### Definition of Sarcopenia

In this study, cut-off values recommended by AWGS 2019 were utilized for identifying low calf circumference (males: < 34 cm and females: < 33 cm), low SMI (males: < 7.0 kg/m^2^ and females: < 5.7 kg/m^2^), low HGS (males: < 28 kg and females: < 18 kg), and low SPPB score (total score ≤ 9 for both sexes) [1]. Calf circumference cut-off values were used for screening or case-finding of sarcopenia. Sarcopenia was defined as cases with both low muscle mass and strength (low SMI + low HGS), irrespective of the SPPB score, and cases with poor physical performance were classified into severe sarcopenia (low SMI + low HGS + low SPPB score) [1].

### Patient demographics and clinical data

Demographic information, pain-related data, and clinical data were extracted from the institutional electronic medical record database system. Patient characteristics encompassed age, sex, and body mass index (BMI). Patient history of diagnosed comorbid conditions and current medications was obtained. Conditions assessed included fall history, cerebro-cardiovascular diseases, diabetes mellitus, osteoporosis, and urinary incontinence. The presence of leg pain (a sciatica symptom), pain duration, and average pain intensity score using a 0 to 10 numeric rating scale (NRS) for the preceding week were identified as pain-related variables.

### Statistical analysis

Descriptive statistics were utilized to summarize continuous variables and are presented as mean values along with standard deviations (SD) and ranges. Categorical variables are expressed as counts and percentages. For data not conforming to normal distribution, median values and interquartile ranges (IQR) are reported with the Shapiro-Wilk test normality assessment results. To compare patient characteristics between the non-sarcopenia and sarcopenia groups, various statistical tests were employed. Independent Student’s t-tests compared means for continuous variables with normal distributions, while the Mann–Whitney U test compared medians for continuous variables with non-normal distributions. Chi-squared tests or Fisher’s exact tests were used for categorical variables. To explore the relationship between calf circumference and SMI, HGS, and SPPB score, linear regression analysis was performed with adjustments for age, BMI and comorbidities that showed significant differences between sarcopenia and non-sarcopenia groups. Specifically, calf circumference was adjusted based on BMI categories (< 25 kg/m² [normal], 25–29 kg/m² [overweight], and ≥ 30 kg/m² [obese]), as recommended by Gonzalez et al. [22], to address potential underestimation in individuals with excess weight who could otherwise show falsely normal calf circumference values. Receiver operating characteristic (ROC) curve analysis was utilized to assess the predictive ability of calf circumference, and corresponding area under the curve (AUC) values and confidence intervals were calculated. Sex-specific calf circumference cut-off values for predicting low SMI, sarcopenia, and severe sarcopenia were determined using ROC analysis and the Youden index. Statistical analyses were conducted using IBM SPSS Statistics, version 25.0 (IBM Corp, Armonk, NY), and statistical significance was set at a *p* -value less than 0.05.

## Results

**Fig. 1:**
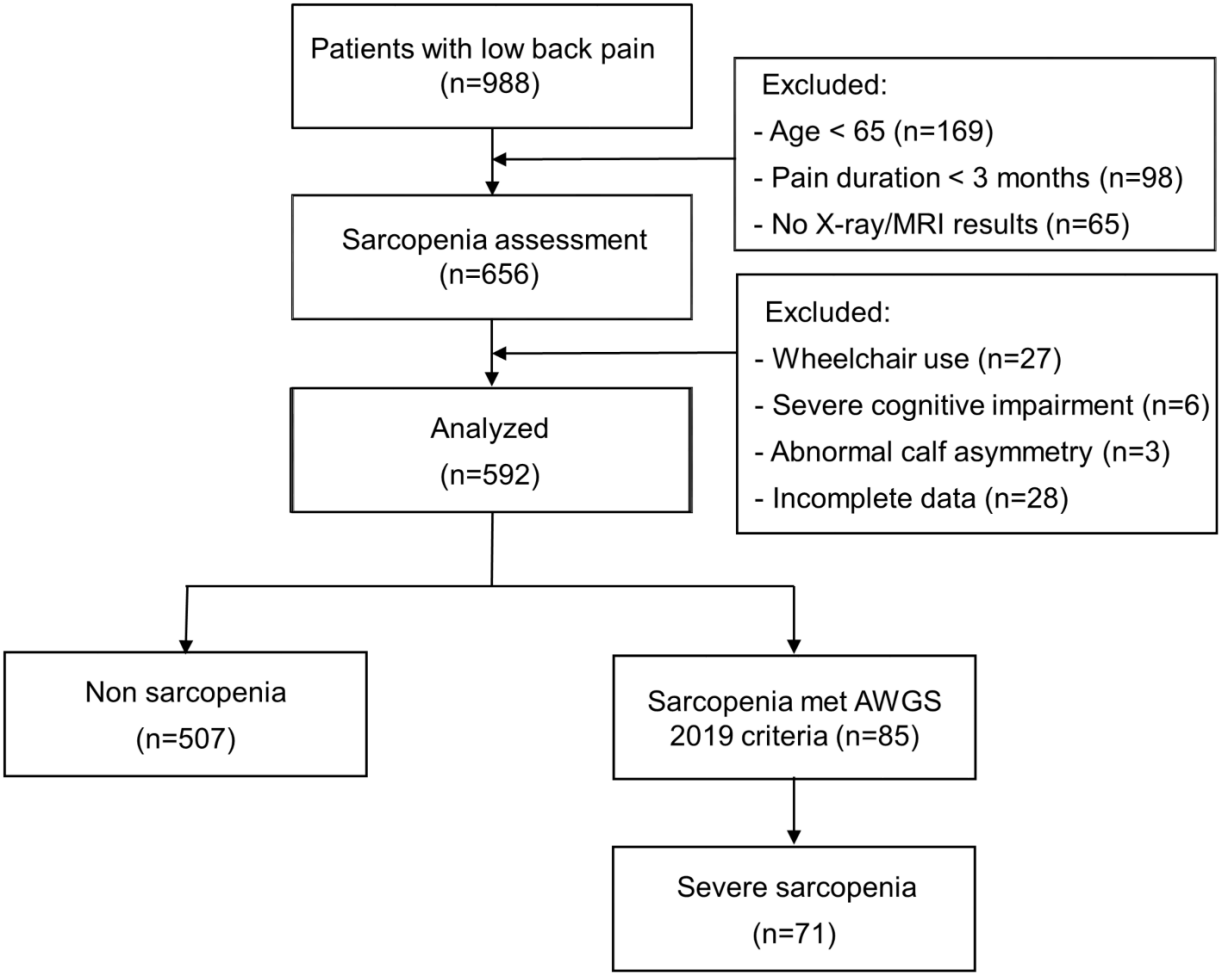
Study flowchart. MRI, magnetic resonance imaging; AWGS, Asian Working Group for Sarcopenia

Within the study period, 988 patients presented with low back pain as their chief complaint at our clinic. After excluding 396 patients based on the study’s exclusion criteria, 592 patients aged 65–90 years (mean age 71.77 ± 6.24 years) were included in the analysis. The sample consisted of 187 males and 405 females. All participants underwent sarcopenia assessment according to the AWGS 2019 criteria, with 507 patients classified as non-sarcopenic and 85 patients (14.3%) classified as sarcopenic (Fig. 1). There was a notable difference in the prevalence of sarcopenia between male and female patients; prevalence was 9.0% among males and 16.7% among females (*p* = 0.016). The number of patients diagnosed as having severe sarcopenia was 71 out of 592 patients (11.9%).

A comparison of patient demographics, comorbid medical conditions, sarcopenia-related measurements, and pain-related data between patients with and without sarcopenia is presented in Table 1. For both sexes, older patients and patients with lower BMIs were more frequently diagnosed with sarcopenia. In the sarcopenia group, more patients of both sexes had a history of falling. The prevalence of osteoporosis was higher in women with sarcopenia than in those without sarcopenia. Smaller calf circumference, lower muscle mass, lower HGS, and lower SPBB scores were observed in the sarcopenia group. Between the two groups in both sexes, there were no significant differences in pain-related variables. Also, after adjusting for age, BMI, and comorbidities, calf circumference showed a positive correlation with SMI but not with HGS and SPPB score in both male and female patients (Table 2).

**Table 1:** Comparing demographics, comorbidities, sarcopenia measures, and pain data between those with and without Sarcopenia

Variables	Males			Females		
	No sarcopenia (*n* = 170)	Sarcopenia (*n* = 17)	*p-*value	No sarcopenia (*n* = 337)	Sarcopenia (*n* = 68)	*p-*value
*Demographic data*						
Age, years	71.70 ± 6.26 (65–89)	75.53 ± 8.23 (65–90)	0.079	71.17 ± 5.87 (65–89)	74.03 ± 6.69 (65–89)	< 0.001
BMI, kg/m^2^	25.15 (23.39;27.46)	23.30 (20.68;24.67)	0.003	25.47 (23.34;27.70)	22.05 (20.56;24.14)	< 0.001
< 25, n	81 (47.6)	13 (76.5)	0.044	152 (45.1)	55 (80.9)	< 0.001
≥ 25, n	89 (52.4)	4 (23.5)		185 (54.9)	13 (19.1)	
Medical comorbidities, n						
Fall	35 (20.6)	8 (47.1)	0.030	87 (25.9)	29 (42.6)	0.008
Cerebro- cardiovascular diseases	57 (33.5)	5 (29.4)	0.941	91 (27.1)	13 (19.1)	0.223
Diabetes	42 (24.7)	5 (29.4)	0.894	87 (25.9)	23 (33.8)	0.234
Osteoporosis	4 (2.4)	1 (5.9)	0.943	82 (24.4)	27 (39.7)	0.015
Urinary incontinence	2 (1.2)	0 (0.0)	> 0.999	58 (17.3)	16 (23.5)	0.295
*Sarcopenia-related data*						
Calf circumference, cm	34.75 ± 2.91	32.12 ± 2.67	< 0.001	33.07 ± 3.03	30.78 ± 2.49	< 0.001
Low calf circumference, n	74 (43.6)	14 (82.4)	0.002	144 (42.7)	51 (75.0)	< 0.001
Normal calf circumference, n	96 (56.5)	3 (17.6)		193 (57.3)	17 (25.0)	
SMI, kg/m^2^	8.08 ± 0.97	6.14 ± 0.50	< 0.001	6.44 ± 1.01	5.03 ± 0.54	< 0.001
HGS, kg	31.54 ± 7.57	21.07 ± 5.22	< 0.001	19.60 ± 4.48	14.15 ± 2.91	< 0.001
SPPB, 0–12	7.49 ± 2.23	5.18 ± 2.70	< 0.001	7.02 ± 1.94	6.57 ± 2.15	0.088
*Pain-related data*						
Leg pain, yes	106 (62.4)	13 (76.5)	0.374	231 (68.5)	52 (76.5)	0.248
Pain duration, months	24.00 (3.00-600.00)	24.00 (3.00-240.00)	0.777	24.00 (3.00-720.00)	36.00 (3.00-480.00)	0.233
< 12 months, n	51 (30.0)	5 (29.4)	> 0.999	118 (35.0)	18 (26.5)	0.222
≥ 12 months, n	119 (70.0)	12 (70.6)		219 (65.0)	50 (73.5)	
Pain score, NRS 0–10	4.58 ± 1.69	4.24 ± 2.08	0.431	4.89 ± 1.95	4.90 ± 2.30	0.971
NRS < 7, n	155 (91.2)	15 (88.2)	> 0.999	283 (84.0)	52 (76.5)	0.188
NRS ≥ 7, n	15 (8.8)	2 (11.8)		54 (16.0)	16 (23.5)	

Values are presented as mean ± standard deviation (SD, range) or median (interquartile range) or number of patients (%). AWGS, Asian Working Group for Sarcopenia; BMI, body mass index; SMI, skeletal muscle mass index; HGS, handgrip strength; SPPB, short physical performance battery; NRS, numeric rating scale

**Table 2:** Correlations between calf circumference and SMI, HGS, and SPPB score adjusted by age, BMI, and comorbidities

	Unadjusted *R*^2^	Adjusted *R*^2^	β	95% CI	*p*-value
Male					
SMI, kg/m^2^	0.375	0.358	1.121	0.745–1.497	< 0.001
HGS, kg	0.270	0.250	0.049	-0.003-0.101	0.065
SPPB, 0–12	0.259	0.239	0.079	-0.096-0.253	0.375
Females					
SMI, kg/m^2^	0.306	0.295	0.661	0.405–0.917	< 0.001
HGS, kg	0.261	0.250	0.014	-0.044-0.072	0.641
SPPB, 0–12	0.265	0.254	-0.112	-0.256-0.032	0.128

BMI, body mass index; SMI, skeletal muscle mass index; HGS, handgrip strength; SPPB, short physical performance battery

**Fig. 2:**
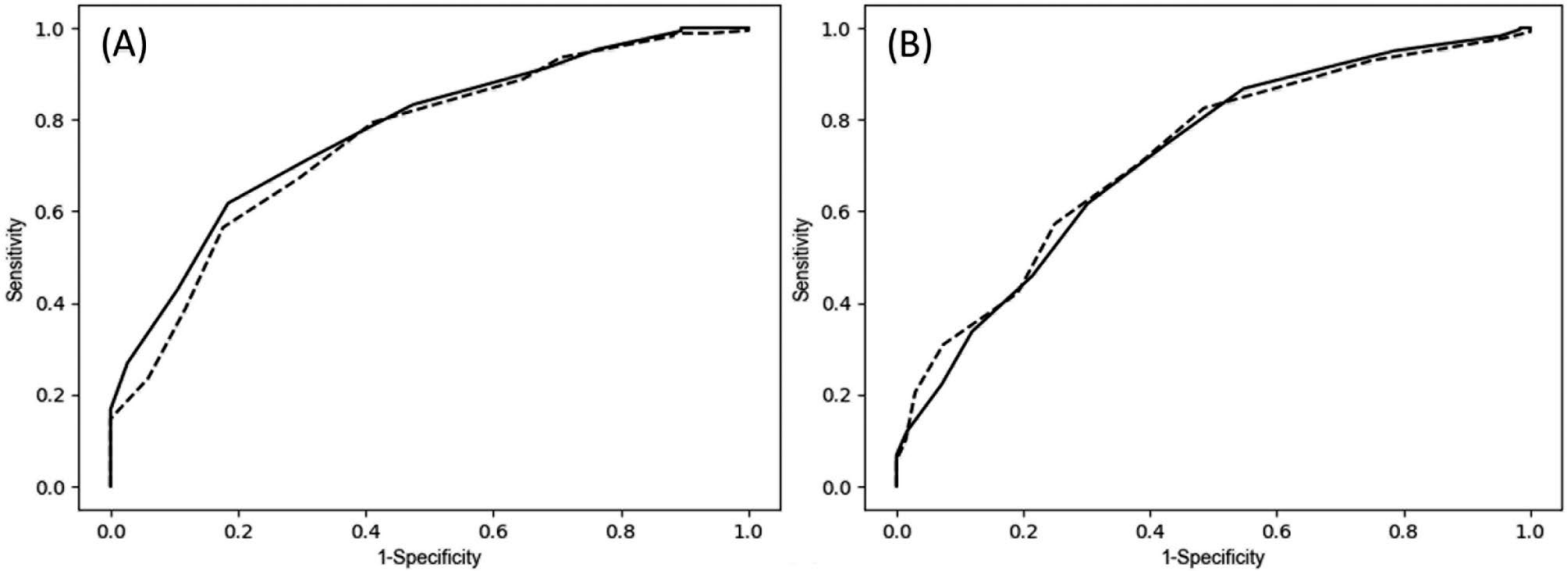
Receiver operating characteristic curves for calf circumference in predicting low muscle mass and sarcopenia. Receiver operating characteristic curves for calf circumference in the prediction of low muscle mass (solid line) and sarcopenia (dotted line) in males **(A)** and females **(B)** The area under the curve values with 95% confidence intervals for low muscle mass and sarcopenia were 0.776 (0.698–0.854) and 0.754 (0.636–0.871), respectively in males, and 0.717 (0.663–0.771) and 0.721 (0.657–0.786), respectively, in females

The results of ROC analysis for predicting low muscle mass and sarcopenia using calf circumference values are illustrated in Fig. 2. The AUC values for low SMI and sarcopenia were 0.776 (95% CI = 0.698–0.854, *p* < 0.001) and 0.754 (95% CI = 0.636–0.871, *p* = 0.001), respectively, in males, and 0.717 (95% CI = 0.663–0.771, *p* < 0.001) and 0.721 (95% CI = 0.657–0.786, *p* < 0.001), respectively, in females. The cut-off values of calf circumference for predicting low SMI and sarcopenia were 34 cm (sensitivity 71.8%, specificity 68.4%) and 34 cm (sensitivity 67.1%, specificity 70.6%), respectively, in males, and 32 cm (sensitivity 74.9%, specificity 57.1%) and 31 cm (sensitivity 82.5%, specificity 51.5%) respectively, in females. When applying the AWGS 2019 cut-off of calf circumference, < 33 cm, for predicting sarcopenia in female patients, sensitivity and specificity were 57.3% and 75.0%, respectively.

## Discussion

In this study, we observed that calf circumference cut-off values for predicting low muscle mass and sarcopenia were determined to be 34 cm in males, while in females, these values were 32 cm and 31 cm, respectively, which diverged from the AWGS 2019 recommendations. Furthermore, our findings indicated a significant positive correlation between calf circumference and muscle mass, though no such association was observed with muscle strength and physical performance measures.

Previously reported cut-offs for calf circumference were 32 to 34 cm in men and 32 to 33 cm in women among the older Asian population [8–12]. These values were developed in consideration of the increase in sensitivity and were consistent with AWGS 2019 recommendations of < 34 cm for men and < 33 cm for women during sarcopenia screening or case-finding [1]. The AUC value of calf circumference cut-offs suggested by AWGS 2019 for predicting sarcopenia (defined by low SMI and low HGS) was 0.647 in 2123 adults aged 70 to 84 years [11]. In 657 adults with mean age of 76.2 years, the AUC values of calf circumference for predicting sarcopenia met AWGS 2019 criteria, 0.82 for men and 0.72 for women [12]. Thus, the predictive performance of calf circumference for sarcopenia in the study population, AUC = 0.754 in males and AUC = 0.721 in females, was similar to previous results from the older population data according to AWGS 2019 criteria. These data are clinically acceptable; however, the predictive power of calf circumference for muscle mass and sarcopenia was lower in women than in men in this study. This observation was consistent with previous results [7, 12]. As higher fat mass in the legs is generally observed in women compared to men [23], this factor could potentially affect the predictive power of calf circumference regarding muscle mass and sarcopenia in female patients in this study.

The pattern of changes in calf circumference in patients with symptomatic degenerative lumbar spinal disease has not been widely studied. Peripheral nerves originating from the lumbar spinal nerves are distributed to the muscles of the lower limbs. In this anatomical context, muscle denervation as the result of neural compression following degenerative change of lumbar spine structures causes a reduction in muscle size in the affected area of the lower limbs [24]. In older patients with CLBP, leg pain and neurogenic claudication can precipitate a detrimental cycle in which reduced physical activity contributes to muscle atrophy and exacerbates deconditioning and disability [25]. Furthermore, electromyographical evidence suggests that reinnervation of muscle fibers in the older population with sarcopenia to compensate for the loss of innervating motor neurons and denervation of muscle fibers was observed significantly less frequently than in healthy controls [26]. These potential changes of calf circumference in our study population might affect relatively lower sensitivity values of calf circumference for identifying low muscle mass and sarcopenia when compared with those from general older papulation data [12].

Also, the clinical features discussed occur more among women with CLBP than among men; in addition, among patients with degenerative lumbar spinal disease, female patients have higher pain scores and more frequent functional impairment and lower quality of life than male patients [27]. In this study, the prevalence of sarcopenia was almost twice as high in female patients compared to male patients. The difference in the prevalence of sarcopenia between sexes varies depending on which guidelines are applied. In recent European and Asian guideline reports, sarcopenia was more prevalent in men than in women [11, 28]. Although the causal relationship between sarcopenia and pain cannot be determined from this study, female patients seem to be more vulnerable to the risk of sarcopenia in chronic pain conditions.

Our results showed that the proposed AWGS 2019 calf circumference cut-off values were valid for predicting sarcopenia in male patients with CLBP. However, in female CLBP patients, the sensitivity of calf circumference for predicting sarcopenia was 82.5% when applying a cut-off of < 31 cm; however, when applying the AWGS 2019 recommended value of < 33 cm, a 30% reduction in sensitivity resulted. Therefore, when using calf circumference as a case-finding marker for sarcopenia among patients with CLBP, sex difference in predictive value for sarcopenia should be considered.

Notably, severe sarcopenia was more prevalent in the study population than in the general older population. In a previous study using AWGS 2019 criteria, the prevalence of severe sarcopenia was 3.3% [11], but our prevalence was 11.9%, almost four times higher. In this study, physical performance was measured using SPPB, a tool designed to evaluate lower limb function encompassing balance, strength, and mobility [21]. We found that the presence or absence of sarcopenia did not correlate with differences in reported pain levels or pain-related characteristics among our study participants. However, it is important to note that patients with CLBP often experience leg or foot pain and may exhibit difficulties in walking, which could adversely impact their SPPB scores. This suggests that while sarcopenia may not directly correlate with reported pain levels, the functional implications of CLBP are significant considerations in this patient population.

Anthropometric measurements do not reflect body composition including intramuscular and subcutaneous fat. Therefore, calf circumference does not fully reflect muscle quality which is closely related to muscle strength and physical function [29]. Indeed, calf circumference did not significantly correlate with muscle strength and physical performance in this study, which contrasts with the results from the general older population [12]. Recent research has indicated that age-related declines in skeletal muscle strength, muscle mass, and muscle quality vary between the upper limbs and lower limbs, leading to potential differences in clinical interpretations for diagnosing sarcopenia [30, 31]. Therefore, when diagnosing sarcopenia and evaluating the severity of sarcopenia for this population, it is crucial to employ a multidimensional assessment approach that considers not only anthropometric measurements and functional assessments but also integrates the clinical characteristics of the chronic pain condition and specific muscle group impairments.

This study has some limitations. The study was conducted at a single tertiary care hospital and included patients of a homogeneous racial and ethnic background, potentially limiting the generalizability of our results to other clinical settings and populations. Our study specifically included patients with confirmed degenerative lumbar spinal diseases identified through radiological evaluation, excluding those with idiopathic low back pain, which is the most prevalent type. This selection criterion may restrict the external validity of our findings. The sample size, particularly for male participants, was small. This not only increases the possibility of sample bias but also limits the statistical power to detect differences and associations accurately within the study cohort. This retrospective analysis only included patients with complete clinical data; the presence of selection biases in the findings cannot be entirely ruled out. The ROC curve can be influenced by class imbalance, where the number of non-sarcopenic cases outweighs the number of sarcopenic cases. This imbalance can lead to misleading optimism about the diagnostic performance of calf circumference as a predictor for sarcopenia. As this study adopts a cross-sectional design, a causal relationship between calf circumference and sarcopenia could not be established. Consequently, longitudinal studies are necessary to validate our findings and elucidate any potential causal associations. BIA is not considered the gold standard for body composition measurement. Also, we did not exclude patients taking diuretic and corticosteroid medications from the analyses, which could affect body water distribution and potentially influence BIA results. However, BIA measurements with multifrequency devices have shown closer correlation with ASM measured by dual-energy X-ray absorptiometry and its adequate performance across multiple domains [32]. Additionally, while there is no worldwide consensus on the exact list of geriatric syndromes, we collected data on several important factors leading to geriatric syndromes, including falls, urinary incontinence, functional decline, and sarcopenia. Although polypharmacy was not explicitly investigated, the comorbidities we examined are based on current medication diagnoses and thus reflect drug administration to some extent. Specific malnutrition and cognitive impairment statuses were not measured with dedicated tools for each individual; however, we excluded patients who were non-ambulatory or unable to complete the sarcopenia assessment due to severe cognitive impairment. Future studies should include a broader range of factors to provide a more comprehensive assessment and to better inform clinical interventions.

## Conclusions

In conclusion, calf circumference appears to be a proxy marker for muscle mass estimated by BIA measurements and may serve as a potential case-finding marker for sarcopenia in older patients with CLBP. Also, although the predictive characteristics differed between the sexes, the predictive performance of calf circumference for sarcopenia in the study population was similar to the results from the older, community-dwelling population data. Therefore, our results suggest that calf circumference is a clinical indicator for predicting muscle mass and may serve as a case-finding marker for sarcopenia in older patients with CLBP.

## Data Availability

The data that support the findings of this study are available from the corresponding author upon reasonable request.
